# Jaungo-based herbal complex regulates psoriasis-associated macrophage–keratinocyte inflammatory axis

**DOI:** 10.3389/fphar.2026.1819017

**Published:** 2026-07-07

**Authors:** Ji-A Byeon, Sang-Kyu Ye, Eun-Bi Seo, Yong-Jin Kwon

**Affiliations:** 1 Department of Cosmeceutical Science, Kyungsung University, Busan, Republic of Korea; 2 Department of Pharmacology, Seoul National University College of Medicine, Seoul, Republic of Korea; 3 Ischemic/Hypoxic Disease Institute, Medical Research Center, Seoul National University, Seoul, Republic of Korea; 4 Department of Cosmetic Science, Kyungsung University, Busan, Republic of Korea

**Keywords:** anti-inflammatory activity, cytokine signaling, epidermal barrier, jaungo-based herbal complex (JBHC), macrophage–keratinocyte inflammatory axis, psoriasis

## Abstract

**Background/Objectives:**

Psoriasis is a chronic inflammatory skin disease characterized by dysregulated cross-talk between macrophages and keratinocytes, resulting in excessive cytokine signaling and impaired epidermal barrier function. Traditional Jaungo has long been used as a topical remedy for inflammatory skin disorders, and its core herbal components exhibit well-documented anti-inflammatory and tissue-protective properties. This study aimed to develop a Jaungo-based herbal complex (JBHC) and evaluate its effects on macrophage-mediated inflammatory responses and keratinocyte barrier dysfunction under psoriasis-like conditions.

**Methods:**

Publicly available transcriptomic datasets were analyzed to identify inflammatory and barrier-related gene expression patterns in psoriatic lesions. The anti-inflammatory effects of JBHC were assessed in LPS-stimulated RAW264.7 macrophages by analyzing the expression of inflammatory mediators and signaling pathways. To evaluate macrophage–keratinocyte cross-talk, conditioned media from activated macrophages, with or without JBHC treatment, were applied to keratinocytes, followed by analysis of epidermal barrier gene expression.

**Results:**

Transcriptomic analyses revealed consistent upregulation of inflammatory cytokines and chemokines, accompanied by significant suppression of key epidermal barrier genes in psoriatic lesions. JBHC markedly attenuated LPS-induced inflammatory signaling in RAW264.7 macrophages by downregulating NOS2, PTGS2, and multiple cytokine genes, while selectively modulating STAT1/STAT3 activation. Conditioned media from activated macrophages significantly suppressed the expression of filaggrin (FLG) and loricrin (LOR) in keratinocytes; however, conditioned media from JBHC-treated macrophages effectively mitigated this barrier gene suppression.

**Conclusion:**

JBHC modulates macrophage-mediated inflammatory pathways and protects keratinocyte barrier gene expression under psoriasis-like inflammatory conditions. These findings suggest that JBHC has potential as a functional herbal complex targeting the macrophage–keratinocyte inflammatory axis in chronic inflammatory skin diseases such as psoriasis.

## Graphical Abstract

**Figure:**
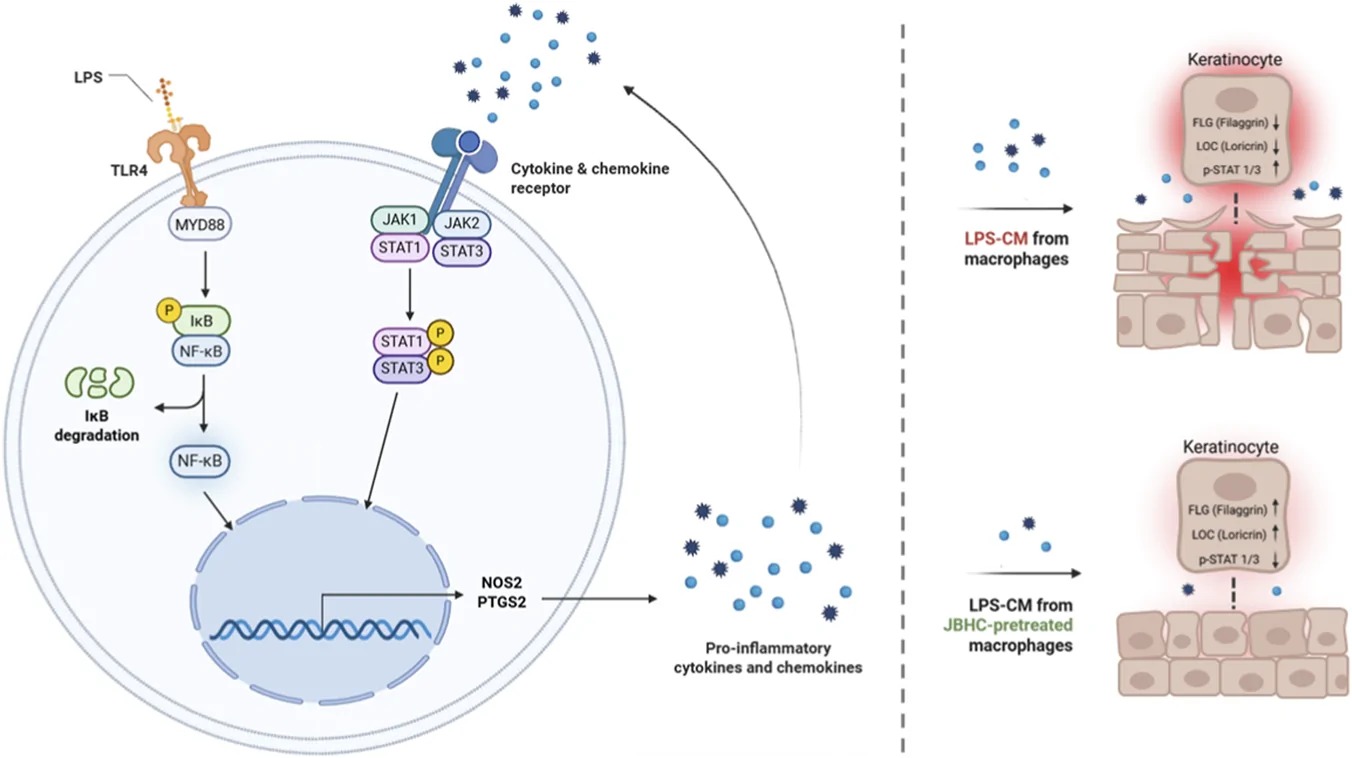

## 1 Introduction

Inflammation is an essential immune response activated in response to external stimuli or pathogen invasion (Medzhitov, 2008). However, excessive activation of macrophages can alter gene expression and cell function in adjacent cells, ultimately disrupting tissue homeostasis (Chen et al., 2023). Pro-inflammatory stimuli, including lipopolysaccharide (LPS), induce macrophages to produce cytokines such as Interleukin (IL)-1β, IL-6, Tumor Necrosis Factor (TNF)-α, and nitric oxide (NO) (Marrocco and Ortiz, 2022). These inflammatory mediators are known to suppress keratinocyte differentiation and downregulate genes important for epidermal barrier formation (Kim et al., 2011; Gutowska-Owsiak et al., 2012).

In inflammatory skin disorders such as psoriasis, these macrophage-derived inflammatory signals are accompanied by reduced expression of barrier-forming proteins, including filaggrin (FLG) and loricrin (LOR) (Kim et al., 2011; Orsmond et al., 2021). This pattern indicates that macrophage-secreted mediators can directly disrupt epidermal barrier integrity (Orsmond et al., 2021). Prolonged exposure to cytokines and reactive mediators secreted by activated macrophages further disrupts keratinocyte differentiation programs and reduces the expression of genes required for proper barrier formation (Gutowska-Owsiak et al., 2012; Orsmond et al., 2021). Consequently, chronic inflammatory conditions further exacerbate barrier dysfunction.

The interaction between macrophages and keratinocytes plays a pivotal role in amplifying inflammatory responses in skin diseases (Kamata and Tada, 2023; Jiang et al., 2024; Nazimek and Bryniarski, 2024). In particular, inflammatory mediators secreted by activated macrophages directly suppress the expression of genes related to barrier and differentiation in keratinocytes (Kamata and Tada, 2023; Jiang et al., 2024). This interaction forms the macrophage–keratinocyte inflammatory axis, maintaining an inflammatory microenvironment and forming a key pathogenic network that contributes to epidermal barrier impairment (Kamata and Tada, 2023; Nazimek and Bryniarski, 2024).

Macrophage-derived inflammatory signaling activates multiple downstream pathways, including Signal Transducer and Activator of Transcription (STAT)-1 and STAT-3, which further amplify inflammatory responses by increasing the expression of inflammatory enzymes such as inducible Nitric Oxide Synthase (iNOS) and Cyclooxygenase (COX)-2 (Lai et al., 2009; Kim et al., 2019). Therefore, modulation of STAT signaling in macrophage is an important regulatory mechanism for suppressing excessive inflammatory mediator production and reducing inflammatory stress transmitted to keratinocytes (Ma et al., 2023). The LPS-induced RAW264.7 macrophage model is widely utilized to experimentally assess such inflammatory signaling events and to characterize the macrophage–keratinocyte axis (Wei et al., 2022; Li et al., 2025).

Jaungo (紫雲膏) is a traditional external herbal formula composed of *Lithospermum erythrorhizon* Siebold & Zucc. and *Angelica sinensis*, historically used for soothing the skin, reducing inflammation, and promoting tissue protection (Ahn et al., 2018; Kim et al., 2023). The anti-inflammatory and protective effects of *L. erythrorhizon* and the antioxidant and tissue-supportive properties of *A. sinensis* have been documented in numerous studies (Han et al., 2008; Saw et al., 2013; Zhuang et al., 2016; Oh et al., 2021). However, despite its long-standing topical use, investigations into herbal complexes derived from traditional Jaungo that can regulate macrophage–keratinocyte inflammatory interactions remain limited. In particular, the potential of such formulations to modulate macrophage-derived inflammatory signaling and its downstream effects on keratinocyte barrier function has not been sufficiently elucidated. Moreover, whether modification of the traditional formulation can enhance its regulatory effects on this inflammatory axis remains largely unexplored.

In this study, we developed an extended herbal complex, termed the Jaungo-based Herbal Complex (JBHC), by supplementing the traditional Jaungo composition with an extract of *Astragalus membranaceus* Bunge. This botanical drug is well known for its immunomodulatory and anti-inflammatory activities and was incorporated to expand the functional scope of the traditional formulation (Auyeung et al., 2016). Therefore, we aimed to evaluate whether JBHC attenuates macrophage-derived inflammatory signals, restores keratinocyte barrier gene expression suppressed by macrophage-conditioned environments, and modulates the psoriasis-associated macrophage–keratinocyte inflammatory axis.

## 2 Materials and methods

### 2.1 Reagents and antibodies

MTT (3-(4,5-dimethylthiazol-2-yl)-2,5-diphenyltetrazolium bromide) reagent was purchased from Duchefa Biochemie (Haarlem, Netherlands). Lipopolysaccharide (LPS) and Griess reagent were purchased from Sigma-Aldrich (St. Louis, MO, United States of America). Crystal violet was purchased from Daejung Chemicals & Metals Co., Ltd. (Siheung, Republic of Korea). RNAiso Plus reagent was purchased from Takara Bio Inc. (Shiga, Japan). ReverTra Ace™ qPCR RT Master Mix was purchased from Toyobo Co., Ltd. (Osaka, Japan), and 2X ZV GREEN SY qPCR Master Mix was purchased from Zenvia (Seongnam, Re-public of Korea). Anti-p-NFκB, p-IκBα, IκBα, p-STAT-1, STAT-1, p-STAT-3, STAT-3, and COX-2 antibodies were purchased from Cell Signaling Technology (Danvers, MA, United States of America). Anti-iNOS antibody was purchased from Invitrogen (Carlsbad, CA, United States of America), and NFκB antibody was purchased from Santa Cruz Biotechnology (Santa Cruz, CA, United States of America). Horseradish peroxidase (HRP)-tagged secondary mouse and rabbit antibodies were purchased from Enzo Life Science (Farmingdale, NY, United States of America). The catalog numbers of all antibodies are summarized in Supplementary Table 1.

### 2.2 Extraction process of herbal extracts

The plant names of *Lithospermum erythrorhizon* Siebold & Zucc. *(L. erythrorhizon)*, *Angelica gigas* Nakai *(A. gigas)*, and *Astragalus membranaceus* Bunge *(A. membranaceus)* have been checked with https://wfoplantlist.org/on February 27. The plants used in this study were obtained from Hamil Korean Medicine Clinic External Herbal Dispensary (Busan, Republic of Korea). Each material is routinely authenticated according to the specifications of the Korean Pharmacopoeia, including macroscopic and organoleptic evaluation. The voucher specimens of the original plant materials have been deposited and are currently maintained at the Department of Cosmetic Science, Kyungsung University for future reference. The materials were extracted using the same extraction method as in our previous studies (Kwon et al., 2022; Kwon et al., 2023; Kim et al., 2026). Briefly, each dried material was extracted three times with 70% ethanol (EtOH) at room temperature (RT) for 24 h. The resulting supernatants were collected and subjected to filtration to remove insoluble residues. The filtrates were subsequently concentrated under reduced pressure and freeze-dried to yield powdered extracts. The final extracts were designated as *L. erythrorhizon* 70% EtOH extract (LE70E), *A. gigas* 70% EtOH extract (AG70E), and *A. membranaceus* 70% EtOH extract (AM70E), respectively.

### 2.3 High-performance liquid chromatography (HPLC) fingerprint analysis

The sample solution was prepared by dissolving the extracts in methanol at a concentration of 100 mg/mL, followed by filtration through a 0.22 μm syringe filter. HPLC fingerprint analysis was performed using an Agilent Eclipse XDB-C18 column (4.6 × 250 mm, 5 μm) under the conditions summarized in Supplementary Table 2. The mobile phases consisted of solvent A (DIW) and solvent B (methanol), and separation was carried out at 30 °C with a flow rate of 1.0 mL/min. A UV-Vis detector was used at a wavelength of 380 nm, and a 20 μL aliquot of the sample solution was injected for analysis. Detailed HPLC conditions were summarized in Supplementary Table 2.

### 2.4 Cell culture

RAW264.7 (mouse macrophage cell) and HaCaT (human keratinocyte) were obtained from the American Type Culture Collection (ATCC, Manassas, VA, United States of America), and Kera308 (mouse keratinocyte) was obtained from the Cytion GmbH (Eppelheim, Germany). All cell lines used in this study are adherent cells. Cells were cultured in DMEM (Bandio Co., Ltd., Pocheon, Republic of Korea) or RPMI (Bandio Co., Ltd.) containing 1% penicillin/streptomycin (Capricorn Scientific GmbH) and 10% fetal bovine serum (FBS, Bandio Co., Ltd.). Theses cell lines were maintained in a CO_2_ incubator (5% CO_2_, 37 °C and humidified atmosphere, PHCbi, Tokyo, Japan).

### 2.5 Conditioned medium (CM)

RAW264.7 (2 × 10^6^ cells/well) cells were seeded in 6-well plates and incubated overnight in a humidified CO_2_ incubator (PHCbi, Tokyo, Japan). On the following day, the cells were pre-treated with each extract for 2 h and subsequently treated with LPS for 4 h. After LPS exposure, the medium was removed, replaced with fresh culture medium, and the cells were further incubated for 8 h. The collected conditioned media were then applied to Kera308 cells for 24 h.

### 2.6 Cytotoxicity assay

RAW 264.7 (1 × 10^5^ cells/well), HaCaT (1 × 10^4^ cells/well), and Kera-308 (1.5 × 10^4^ cells/well) cells were seeded in 96-well plates and incubated overnight in a humidified CO_2_ incubator (PHCbi). On the following day, the cells were treated with each extract in a dose-dependent manner and further incubated for 24 h. To assess cell viability, MTT reagent was added and incubated for 2 h at 37 °C. The resulting violet formazan crystals were dissolved in dimethyl sulfoxide (DMSO), and absorbance was determined using a microplate reader (BioTek Instruments, Winooski, VT, United States of America) at 570 nm wavelength.

### 2.7 Crystal violet staining

RAW 264.7 (5 × 10^5^ cells/well), HaCaT (1 × 10^5^ cells/well), and Kera-308 (1.5 × 10^5^ cells/well) cells were seeded in 24-well plates and incubated overnight in a humidified CO_2_ incubator (PHCbi). On the following day, the cells were treated with each extract in a dose-dependent manner and further incubated for 24 h. After incubation, the cells were stained for 10 min at RT with 0.5% crystal violet (Daejung Chemicals & Metals Co., Ltd.) dissolved in 20% methanol.

### 2.8 Nitric oxide (NO) production

RAW264.7 (2 × 10^6^ cells/well) cells were seeded in 6-well plates and incubated overnight in a humidified CO_2_ incubator (PHCbi). On the following day, the cells were pre-treated with each extract for 2 h, followed by treatment with LPS for 16 h in a humidified CO_2_ incubator. The culture media were mixed with Griess reagent (Sigma-Aldrich) and incubated at RT for 10 min. Absorbance was determined using a microplate reader (BioTek Instruments) at 570 nm wavelength.

### 2.9 Protein extraction and Western blotting

Cells were washed twice with PBS and lysed using Triton-X lysis buffer containing protease inhibitor cocktail (Abbkine Scientific Co., Ltd., Wuhan, China). The lysates were incubated on ice for 10 min, and then centrifuged at 12,000 rpm for 10 min at 4 °C. The supernatants were collected, and protein concentrations were determined using Bradford reagent (Bio-Rad, Hercules, CA, United States of America). Equal amounts of protein (50 μg per lane) were separated by 10% SDS–polyacrylamide gel electrophoresis (SDS–PAGE) and transferred onto nitrocellulose membranes (GE Healthcare, Chicago, IL, United States of America). The membranes were blocked with 5% skim milk for 1 h at room temperature, followed by incubation with primary antibodies overnight at 4 °C. After washing with Tris-buffered saline containing 0.1% Tween 20 (TBST), the membranes were incubated with horseradish peroxidase (HRP)-conjugated secondary antibodies for 1 h at RT. Protein bands were visualized using enhanced chemiluminescence (ECL) reagents, and images were captured with a Davinch-Chemi™ Imaging System (Davinch-K Co., Ltd., Seoul, Republic of Korea).

### 2.10 RNA extraction and quantitative real time PCR (q-PCR)

Total RNA was extracted using RNAiso Plus reagent (Takara), and complementary DNA (cDNA) was synthesized using the ReverTra Ace® qPCR RT Master Mix (Toyobo) according to the manufacturer’s instruction (https://www.toyobo-global.com/). qRT-PCR was performed with 2X ZV GREEN SY qPCR Master Mix (ZENVIA, Busan, Republic of Korea) on a LightCycler® 96 System (Roche, Basel, Switzerland). All primer sequences were synthesized by Bioneer (Dae-jeon, Republic of Korea), and the mouse and human primer sets used in this study are listed in Supplementary Table 2.

### 2.11 Transcriptomic big data analysis

In this study, publicly available microarray datasets (GSE154200, GSE160086, and GSE298073) were retrieved from the NCBI GEO database (https://www.ncbi.nlm.nih.gov/geo/). Heatmap visualization was performed to compare the transcriptional profiles across different sample groups by normalizing gene expression values using z-scores calculated from the mean and standard deviation of each gene across all samples. Gene set enrichment analysis (GSEA) was carried out using hallmark gene sets from the MSigDB portal (https://www.gsea-msigdb.org) to explore transcriptional differences between lesional and non-lesional skin samples of psoriasis patients included in the GSE154200 dataset.

### 2.12 Statistical analysis

All statistical analyses were conducted using Microsoft Excel (Microsoft Corp., Redmond, WA, United States of America) and GraphPad Prism version 5.0 (GraphPad Software, San Diego, CA, United States of America). Data are expressed as the mean ± standard deviation (SD) obtained from at least three independent experiments. Statistical analysis was performed using an unpaired Student’s t-test for comparisons between two groups, and one-way analysis of variance (ANOVA) followed by Tukey’s multiple comparisons test for comparisons among three or more groups. Statistical significance was defined at *p < 0.05, p < 0.01*, and *p < 0.001*.

## 3 Results

### 3.1 Preparation, characterization, and Cytotoxicity Evaluation of Jaungo-Based Herbal Extracts

**FIGURE 1:**
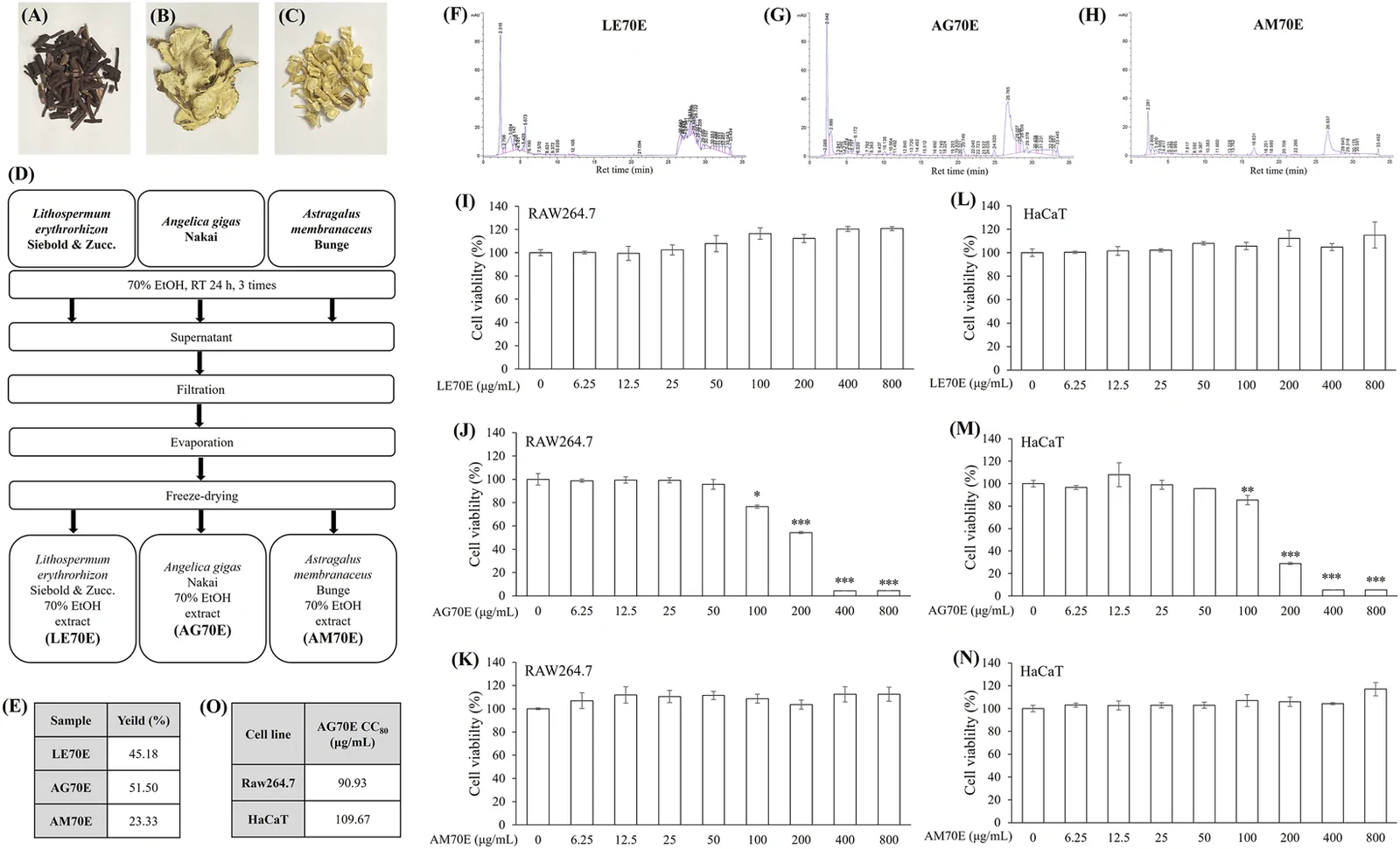
Preparation, Characterization, and Cytotoxicity Evaluation of Jaungo-Based Herbal Extracts. **(A–C)** Raw materials of *Lithospermum erythrorhizon* Siebold & Zucc. **(A)**, *Angelica gigas* Nakai **(B)**, and *Astragalus membranaceus* Bunge **(C). (D)** Extraction process of the herbal extracts using 70% EtOH. **(E)** Extraction yields of herbal extracts. **(F–H)** HPLC fingerprint analysis of LE70E **(F)**, AG70E **(G)**, and AM70E **(H). (I–K)** Cytotoxicity of LE70 E **(I)**, AG70E **(J)**, and AM70E **(K)** in RAW264.7 cells after 24 h of treatment, as evaluated by the MTT assay. **(L-N)** Cytotoxicity of LE70E **(L)**, AG70E **(M)**, and AM70E **(N)** in HaCaT cells after 24 h of treatment, as evaluated by the MTT assay. **(O)** CC_80_ (Cytotoxic Concentration 80%) of AG70E. Data are presented as the mean ± SD from at least three independent experiments. Statistical significance was determined at *p* < 0.05.

Dried *L. erythrorhizon, A. gigas*, and *A. membranaceus* were extracted with 70% ethanol for 24 h and concentrated to obtain LE70E, AG70E, and AM70E, respectively (Figures 1A–D). The extraction yields were 45.18% for LE70E, 51.50% for AG70E, and 23.33% for AM70E (Figure 1E). The phytochemical profiles of the extracts were evaluated through HPLC fingerprint analysis under identical analytical conditions (Figures 1F–H; Supplementary Tables 4–6).

Cytotoxicity was first evaluated in RAW264.7 macrophages (Figures 1I–K). LE70E and AM70E showed minimal cytotoxicity across the tested concentrations, whereas AG70E exhibited a more pronounced, concentration-dependent reduction in cell viability. Similar patterns were observed in HaCaT keratinocytes (Figures 1L–N). LE70E and AM70E showed negligible cytotoxic effects, while AG70E again demonstrated relatively higher cytotoxicity compared with the other extracts. The CC_80_ value of AG70E was calculated to be 90.93 μg/mL in RAW264.7 cells and 109.67 μg/mL in HaCaT cells, confirming its relatively higher cytotoxicity compared to LE70E and AM70E (Figure 1O).

### 3.2 Protective effect of JBHC in LPS-Stimulated RAW264.7 Cells Under Keratinocyte-Compatible Conditions

JBHC used in this study was formulated based on the traditional composition of Jaungo by combining LE70E and AG70E at a 1:1 ratio, with an additional 10% AM70E extract, resulting in a final ratio of 4.5 : 4.5 : 1 (LE70E : AG70E : AM70E).

**FIGURE 2:**
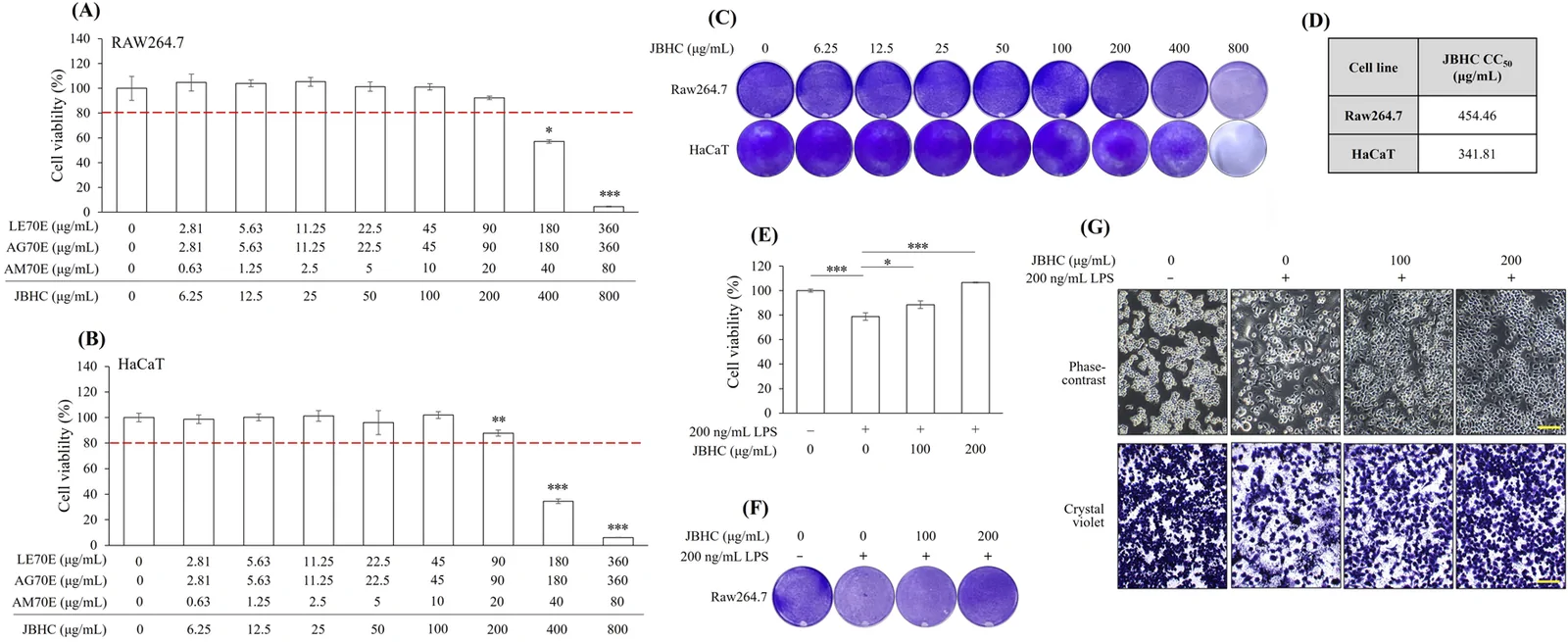
Protective effect of JBHC in LPS-Stimulated RAW264.7 Cells Under Keratinocyte-Compatible Conditions. **(A,B)** Cytotoxicity of JBHC in RAW264.7 **(A)** and HaCaT **(B)** cells after 24 h of treatment, as evaluated by the MTT assay. **(C)** Cytotoxicity of JBHC in RAW264.7 and HaCaT cells after 24 h of treatment, as evaluated by the crystal violet staining. **(D)** CC_50_ (Cytotoxic Concentration 50%) of JBHC. **(E–G)** RAW264.7 cells were pretreated with JBHC for 2 h and then stimulated with LPS for 16 h. Cell viability was assessed using MTT **(E)** and crystal violet **(F)** assays, and cellular morphology was observed using a microscope **(G)**. Scale bar = 100 μm. Data are presented as the mean ± SD from at least three independent experiments. Statistical significance was determined at *p* < 0.05.

The cytotoxicity of JBHC was assessed using MTT assay after 24-h treatment of RAW264.7 macrophages and HaCaT keratinocytes with the extract (Figures 2A,B). Both cell types maintained over 80% viability at 200 μg/mL, and a similar pattern was observed in crystal violet staining results (Figure 2C). The calculated CC_50_ values were 454.46 μg/mL in RAW264.7 cells and 341.81 μg/mL in HaCaT cells, respectively (Figure 2D).

Next, the protective effect of JBHC was examined in an LPS-induced cytotoxicity model using RAW264.7 cells. LPS treatment markedly reduced cell viability, whereas JBHC pretreatment significantly alleviated this decrease in the MTT assay (Figure 2E). The Protective effect of JBHC was further confirmed by crystal violet staining (Figure 2F). Morphological observation also showed that LPS-induced abnormal cellular features, including swelling and elongation, were substantially reduced in the JBHC-treated group (Figure 2G). These results demonstrate that JBHC confers significant protection against LPS-induced cellular injury while maintaining non-cytotoxicity in keratinocytes.

### 3.3 JBHC selectively suppresses STAT-Dependent Psoriatic Inflammatory Signaling

**FIGURE 3:**
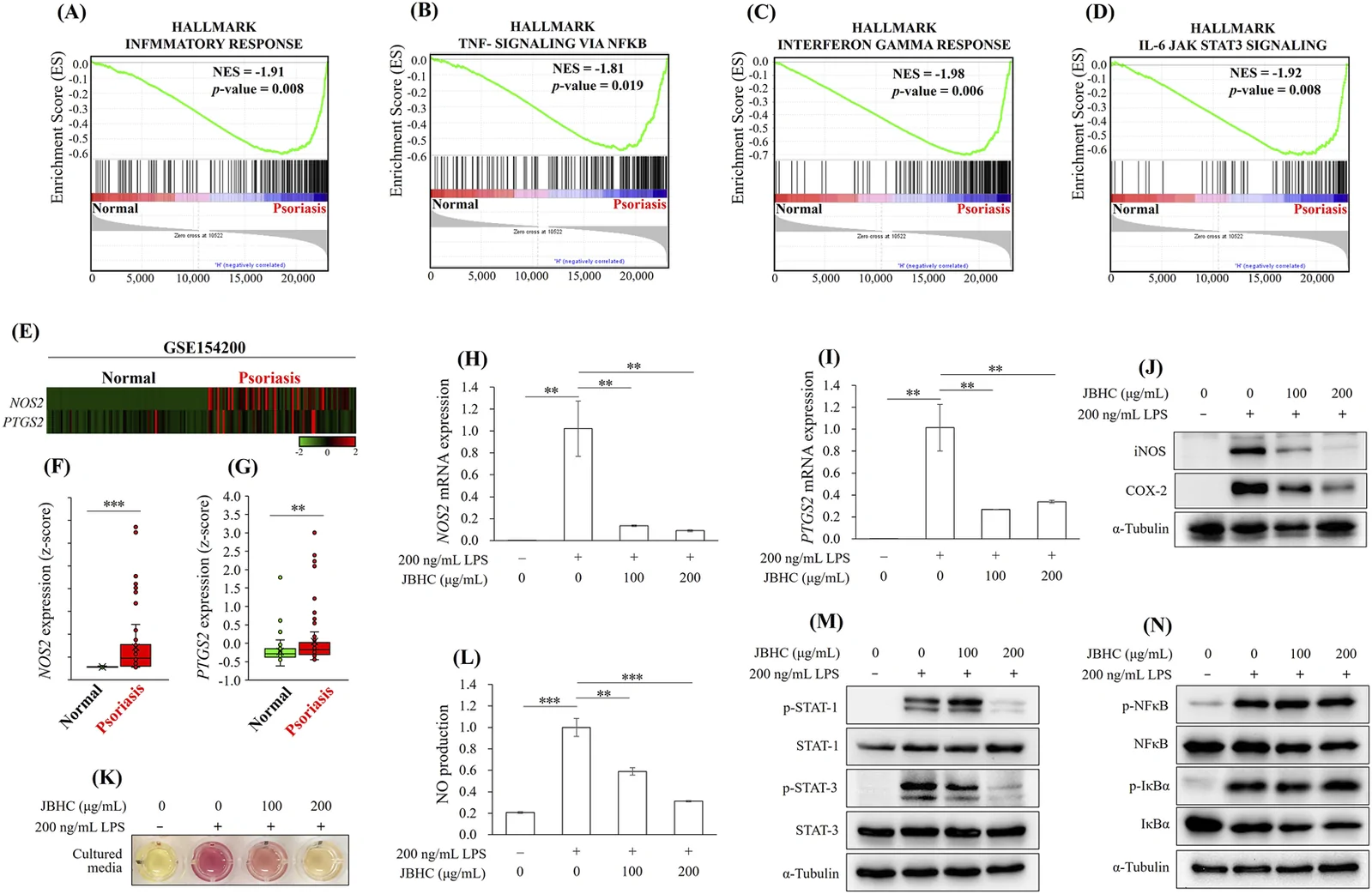
JBHC Selectively Suppresses STAT-Dependent Psoriatic Inflammatory Signaling. **(A–D)** Enrichment plots of hallmark gene sets related to inflammation and cytokine signaling comparing lesional and non-lesional skin of patients with psoriasis **(E–G)** Gene expression analysis, showing differences between lesional and non-lesional skin of patients with psoriasis. Heatmap **(E)** shows the differential expression of NOS2 and PTGS2 between the two groups, and relative expression levels of NOS2 **(F)** and PTGS2 **(G)** in lesional and non-lesional skin of patients with psoriasis from the GEO dataset GSE154200 are displayed as box plots. **(H–L)** RAW264.7 cells were pretreated with JBHC for 2 h and then stimulated with LPS for 16 h. Relative mRNA expression of NOS2 **(H)** and PTGS2 **(I)** was analyzed by qPCR, protein expression of iNOS and COX-2 was determined by Western blot **(J)**, and NO production was measured using the Griess reagent **(K,L). (M,N)** RAW264.7 cells were pretreated with JBHC for 2 h and then stimulated with LPS for 4 h. Protein expression of STAT1/3 **(M)** and NFκB/IκBα **(N)** was determined by Western blot. Data are presented as the mean ± SD from at least three independent experiments. Statistical significance was determined at *p* < 0.05.

GSEA psoriasis lesions versus normal skin revealed significant enrichment of four inflammation-related hallmark gene sets, indicating pronounced activation of inflammatory signaling in psoriatic tissue (Figures 3A–D). Consistent with this, both heatmap visualization and differential expression analysis revealed marked upregulation of NOS2 (iNOS) and PTGS2 (COX-2), key enzymes responsible for NO and PGE_2_ production, in psoriasis samples (Figures 3E–G).

To determine whether JBHC modulates these inflammatory mediators, RAW264.7 macrophages were stimulated with LPS in the presence or absence of JBHC. qPCR analysis showed that JBHC pretreatment substantially reduced LPS-induced mRNA expression of NOS2 and PTGS2 (Figures 3H,I). Western blot analysis confirmed that JBHC inhibited iNOS and COX-2 in LPS-stimulated cells (Figure 3J). In addition, Griess assay results demonstrated that JBHC significantly reduced nitric oxide (Figures 3K,L).

Furthermore, to investigate whether JBHC affects upstream inflammatory signaling mechanisms, we analyzed the activation status of representative pathways involved in macrophage-mediated inflammation. JBHC attenuated LPS-induced phosphorylation of STAT1 and STAT3, but did not significantly inhibit the activation of the NF-κB/IκBα pathway (Figures 3M,N). These results suggest that the anti-inflammatory activity of JBHC is primarily mediated through selective modulation of STAT-dependent signaling rather than through inhibition of NF-κB pathway, which may contribute to the suppression of downstream inflammatory mediators involved in psoriasis.

### 3.4 Integrated Cytokine Signature Analysis and JBHC-Mediated Suppression of Psoriasis-Associated Inflammatory Genes

**FIGURE 4:**
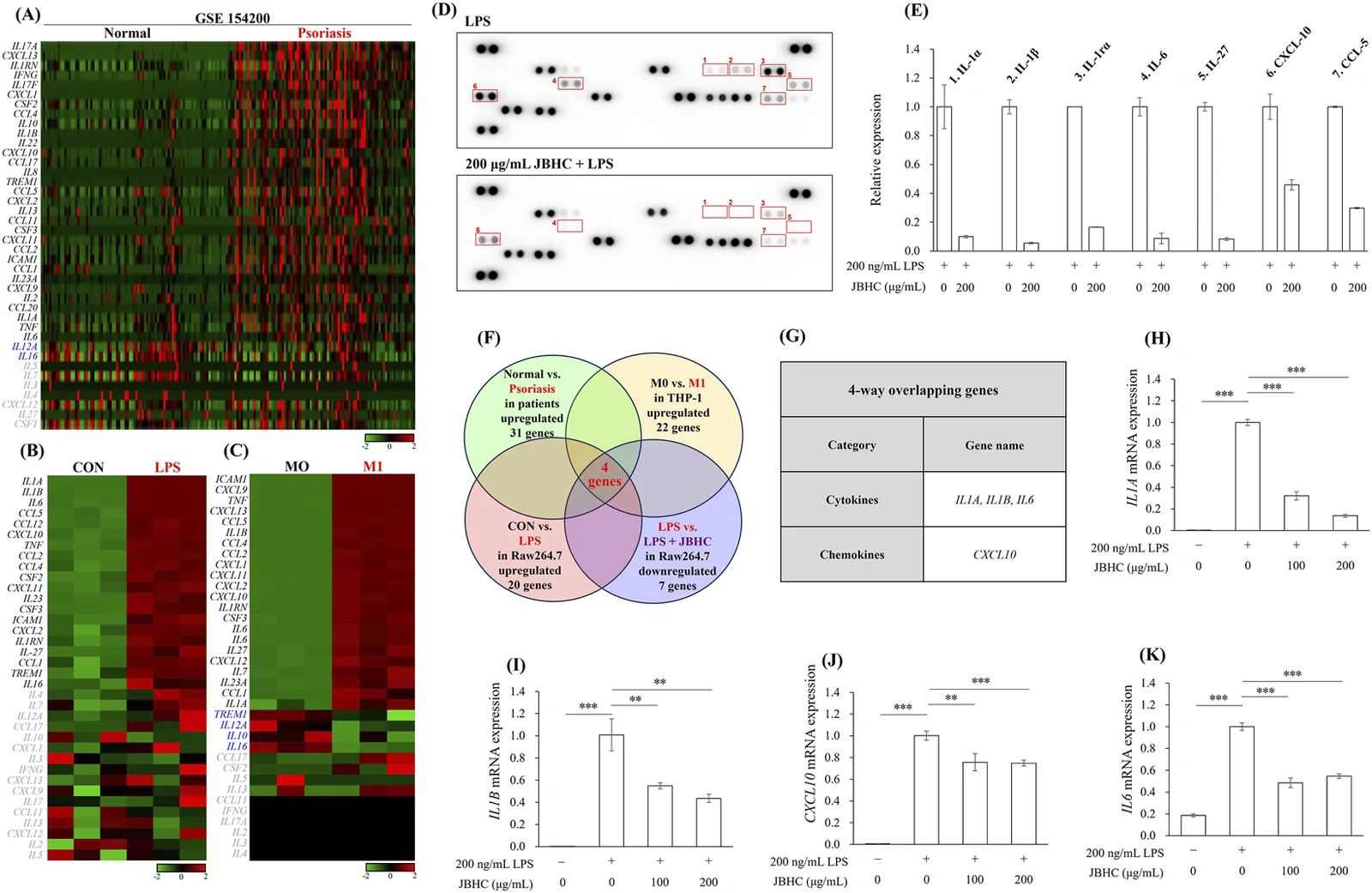
Integrated Cytokine Signature Analysis and JBHC-Mediated Suppression of Psoriasis-Associated Inflammatory Genes. **(A–C)** Heatmap visualization of pro-inflammatory cytokine gene expression from GEO datasets, showing differences under various inflammatory conditions. *GSE154200*: Lesional vs. non-lesional skin of patients with psoriasis **(A)**. *GSE160086*: RAW264.7 macrophages, control (CON) vs. LPS-treated groups **(B)**. *GSE298073*: THP-1 macrophages, M0 vs. M1 polarization states **(C)**. Text color indicates expression trends: black denotes upregulated genes (positive correlation), blue indicates downregulated genes (negative correlation), and gray represents no significant change. **(D,E)** RAW264.7 cells were pretreated with JBHC for 2 h and then stimulated with LPS for 8 h. The cytokines in supernatants were measured using a cytokine array kit **(D)**. The blots of cytokine were quantified using Image**(J)**. The red line indicates a relative decrease in expression of more than half compared to the LPS-treated group **(E). (F,G)** Venn diagram **(F)** and summary table **(G)** showing the overlap of upregulated genes identified in three GEO datasets and downregulated genes in the cytokine array data. **(H–K)** RAW264.7 cells were pretreated with JBHC for 2 h and then stimulated with LPS for 4 h mRNA expression of *IL1A* **(H)**, *IL1B* **(I)**, *CXCL10* **(J)**, *IL6* **(K)**, was analyzed by qPCR. Data are presented as the mean ± SD from at least three independent experiments. Statistical significance was determined at *p* < 0.05.

Integrated transcriptomic analysis was performed using three independent GEO datasets representing different inflammatory contexts: normal vs. psoriatic skin (GSE154200), control vs. LPS-stimulated RAW264.7 macrophages (GSE160086), and M0 vs. M1-polarized THP-1 macrophages (GSE298073). Heatmap profiling revealed consistent upregulation of inflammatory cytokine genes across all three models (Figures 4A–C). Cytokine array analyses showed that LPS-induced increases in IL-1α, IL-1β, IL-1ra, IL-6, IL-27, CXCL-10, and CCL-5 were attenuated by JBHC pretreatment (Figures 4D,E). Venn diagram analysis identified 4 overlapping genes commonly upregulated across these inflammatory contexts, including key cytokines and chemokines (Figures 4F,G).

The regulatory effects of JBHC on these inflammation-related genes were evaluated using LPS-stimulated RAW264.7 macrophages (Figures 4H–K). qPCR analysis demonstrated that JBHC pretreatment markedly reduced LPS-induced expression of IL1A, IL1B and IL6, while CXCL10 exhibited partial but detectable decreases. These results indicate that JBHC selectively suppresses key inflammatory mediators that are commonly upregulated across psoriasis and macrophage activation models, which are known to contribute to macrophage–keratinocyte inflammatory interactions and subsequent barrier dysfunction.

### 3.5 JBHC Protects Keratinocyte Barrier Gene Expression Under Psoriatic Inflammatory Stress

**FIGURE 5:**
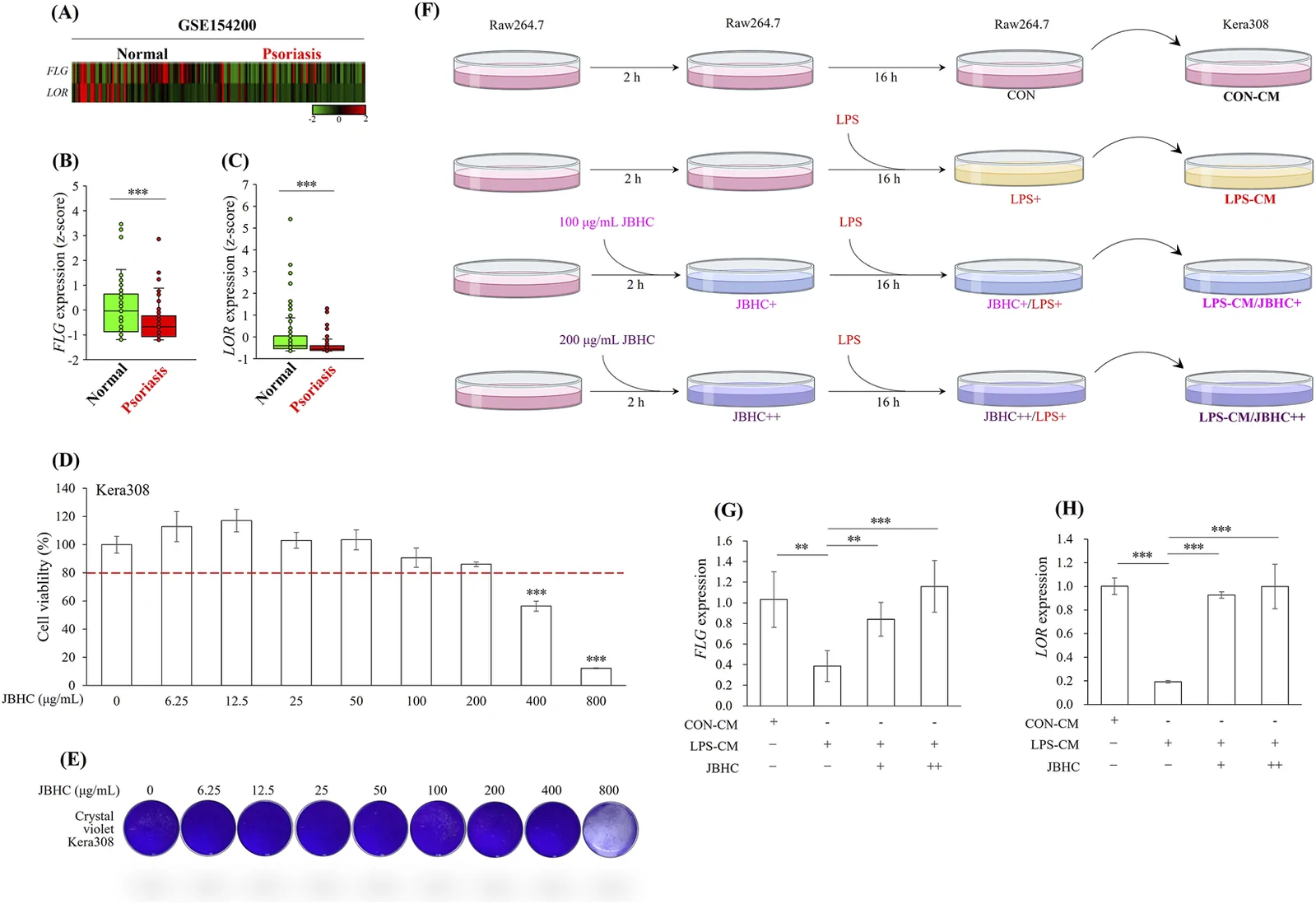
JBHC Protects Keratinocyte Barrier Gene Expression Under Psoriatic Inflammatory Stress. **(A)** Heatmap visualization of genes related to epidermal barrier, differentiation, and proliferation in lesional and non-lesional skin of patients with psoriasis from the GEO dataset GSE154200. **(B,C)** Relative expression levels of FLG **(B)** and LOR **(C)** in lesional and non-lesional skin of patients with psoriasis from the GEO dataset GSE154200 are displayed as box plots. **(D,E)** Cytotoxicity of JBHC in Kera308 cells after 24 h of treatment, as evaluated by MTT **(D)** and crystal violet **(E)** assays. **(F)** RAW264.7 conditioned medium (CM) preparation. **(G,H)** Kera308 cells were treated with each CM for 24 h mRNA expression of FLG **(G)** and LOR **(H)** was analyzed by qPCR. Data are presented as the mean ± SD from at least three independent experiments. Statistical significance was determined at *p* < 0.05.

Psoriatic skin is characterized by severe impairment of the epidermal barrier, and transcriptomic analysis clearly reflected this pathological feature. Heatmap and expression profiling revealed that the key barrier proteins FLG and LOR were significantly downregulated in psoriatic lesions compared with non-lesional skin (Figures 5A–C).

Before assessing barrier responses, the safety of JBHC was assessed in Kera308 keratinocytes. JBHC maintained greater than 80% viability at 200 μg/mL in both MTT and crystal violet assays, demonstrating the feasibility of performing barrier-related experiments at a subtoxic concentration range (Figures 5D,E).

To model macrophage-derived inflammatory stress, conditioned medium (CM) was collected from LPS-stimulated RAW264.7 cells and applied to Kera308 keratinocytes (Figure 5F). Given that macrophage-derived cytokines play a critical role in disrupting keratinocyte barrier function, we next investigated whether JBHC-mediated suppression of inflammatory signaling in macrophages could translate into protective effects in keratinocytes. LPS-CM markedly suppressed the expression of FLG and LOR, whereas CM derived from JBHC-treated macrophages significantly attenuated this reduction (Figures 5G,H). These findings indicate that JBHC effectively counteracts macrophage-mediated suppression of barrier-related genes, suggesting a protective role against epidermal barrier disruption under psoriatic inflammatory conditions.

## 4 Discussion

In this study, we identified the Jaungo-based herbal complex (JBHC) as a functional botanical formulation capable of modulating psoriasis-associated inflammatory pathways and protecting keratinocyte barrier integrity. Our findings demonstrate that JBHC targets the macrophage–keratinocyte inflammatory axis, a pathogenic circuit widely implicated in the initiation and maintenance of psoriatic inflammation (Kamata and Tada, 2023; Jiang et al., 2024). This axis is characterized by chronic activation of macrophages, excessive cytokine production, and downstream suppression of epidermal differentiation programs—features consistently observed in clinical psoriasis as well as in experimental inflammatory models (Stratis et al., 2006; Armstrong and Read, 2020).

A central finding of this study is that JBHC selectively attenuates macrophage-driven inflammatory signaling. Transcriptomic datasets from psoriatic skin and macrophage activation models revealed robust upregulation of pro-inflammatory cytokines, chemokines, and iNOS/COX-2, consistent with previous reports describing the pathogenic role of activated macrophages in psoriasis (Sieminska et al., 2024). JBHC attenuated key features of macrophage-driven inflammatory responses, including excessive nitric oxide production and pro-inflammatory cytokine expression. Mechanistically, this anti-inflammatory activity was associated with selective modulation of STAT1 and STAT3, two transcription factors known to orchestrate macrophage polarization and amplify inflammatory loops in psoriatic lesions (Guo et al., 2023). Importantly, JBHC did not significantly inhibit NF-κB activation, suggesting pathway specificity rather than broad immunosuppression. Such selectivity may provide therapeutic advantages by limiting off-target suppression of essential innate immune functions.

Another major outcome of our study is the demonstration that JBHC protects keratinocyte barrier gene expression under conditions of macrophage-mediated inflammatory stress. Clinical transcriptomic analyses revealed substantial suppression of FLG and LOR in psoriatic lesions, reflecting impaired epidermal differentiation and barrier dysfunction. Our macrophage–keratinocyte co-culture model recapitulated this phenomenon, as conditioned medium from activated macrophages reduced FLG and LOR expression. JBHC-treated macrophage CM effectively mitigated this barrier suppression. These findings suggest that macrophage-derived soluble mediators directly disrupt keratinocyte differentiation programs and that JBHC counteracts this paracrine inflammatory signaling, thereby interrupting a key pathogenic loop linking macrophage activation to barrier impairment in psoriasis. In addition to barrier regulation, keratinocyte STAT3 signaling has been implicated in the pathogenesis of psoriasiform dermatitis (Ravipati et al., 2022). LPS-CM treatment induced STAT3 activation in keratinocytes, and JBHC effectively attenuated this response (Supplementary Figure 2A). To further evaluate the role of STAT3 signaling in barrier gene regulation, the STAT3 inhibitor Nifuroxazide was applied following cytotoxicity assessment, and effective inhibition of STAT3 was confirmed (Supplementary Figure 2B,C). Although STAT3 activation induced by LPS-CM was suppressed, the reduction of FLG and LOR expression was not alleviated (Supplementary Figure 2D,E). These findings suggest that STAT3 signaling alone may not be sufficient to mediate JBHC-induced barrier protection in keratinocytes, and further studies are required to clarify the underlying mechanisms.

The observed biological activities of JBHC are consistent with the known pharmacological properties of its components. *Lithospermum erythrorhizon* Siebold & Zucc. derivatives possess anti-inflammatory and wound-healing effects (Kim et al., 2024; Xue et al., 2024); *Angelica sinensis* extracts exhibit antioxidant and tissue-supportive functions (Zhuang et al., 2016; Hou et al., 2021); and *Astragalus membranaceus* Bunge is widely recognized for immunomodulatory activity targeting macrophage signaling (Li et al., 2018). By combining these herbal extracts, JBHC appears to achieve synergistic attenuation of macrophage hyperactivation while simultaneously enhancing keratinocyte resilience to inflammatory insults. This dual regulatory capacity is particularly relevant in psoriatic pathology, where immune dysregulation and barrier dysfunction reinforce one another to perpetuate chronic inflammation (Orsmond et al., 2021).

Despite these promising observations, several limitations should be considered. First, the current study employed *in vitro* models, and *in vivo* evaluation in imiquimod- or cytokine-induced psoriasis-like mouse models would strengthen the translational relevance. Second, although JBHC modulated STAT1/3 activity, the specific active constituents responsible for this effect remain to be identified; targeted phytochemical profiling and isolation studies are warranted. Finally, the broader influence of JBHC on additional immune cell populations—such as dendritic cells, neutrophils, and T cells—should be explored to fully characterize its immunoregulatory potential.

Overall, our findings position JBHC as a promising herbal complex capable of modulating macrophage-driven inflammatory pathways and preserving epidermal barrier gene expression under psoriatic conditions. By targeting the macrophage–keratinocyte inflammatory axis, JBHC may provide a novel therapeutic approach for managing chronic inflammatory skin diseases characterized by barrier dysfunction and immune dysregulation.

## 5 Conclusion

In this study, we demonstrated that the Jaungo-based herbal complex (JBHC), formulated by combining LE70E and AG70E with supplemental AM70E, exerts anti-inflammatory and barrier-protective activities across macrophage and keratinocyte models relevant to psoriasis. JBHC effectively reduced LPS-induced inflammatory signaling in RAW264.7 macrophages by suppressing key cytokines and chemokines, attenuating NOS2 and PTGS2 expression, decreasing nitric oxide production, and selectively inhibiting STAT1/3 activation. Moreover, macrophage-derived inflammatory factors markedly suppressed keratinocyte barrier genes, mimicking psoriatic conditions, and JBHC significantly alleviated this reduction, restoring FLG and LOR expression. Collectively, these findings demonstrate that JBHC has the capacity to modulate the psoriasis-associated macrophage–keratinocyte inflammatory axis and protect epidermal barrier integrity under inflammatory stress.

## Data Availability

The original contributions presented in the study are included in the article/Supplementary Material, further inquiries can be directed to the corresponding authors.
