# Correction: Jaungo-based herbal complex regulates psoriasis-associated macrophage–keratinocyte inflammatory axis

**DOI:** 10.3389/fphar.2026.1971066

**Published:** 2026-09-30

**Authors:** Ji-A. Byeon, Sang-Kyu Ye, Eun-Bi Seo, Yong-Jin Kwon

**Affiliations:** 1 Department of Cosmeceutical Science, Kyungsung University, Busan, Republic of Korea; 2 Department of Pharmacology, Seoul National University College of Medicine, Seoul, Republic of Korea; 3 Ischemic/ Hypoxic Disease Institute, Medical Research Center, Seoul National University, Seoul, Republic of Korea; 4 Department of Cosmetic Science, Kyungsung University, Busan, Republic of Korea

**Keywords:** anti-inflammatory activity, cytokine signaling, epidermal barrier, jaungo-based herbal complex (JBHC), macrophage–keratinocyte inflammatory axis, psoriasis

Grant/funding/project number incorrect

An incorrect number was provided for [the “Busan Regional Innovation System & Education (RISE)” Project, supported by the Ministry of Education and Busan Metropolitan City. The incorrect project number was **2026-RISE-05-005-001**]. The correct number is [**2026-RISE-05-005-010**].

The original version of this article has been updated.

