# Supplementary material for: Jaungo-based herbal complex regulates psoriasis-associated macrophage–keratinocyte inflammatory axis
Source: Front Pharmacol. 2026 Jul 7;17:1819017. doi: 10.3389/fphar.2026.1819017 (PMC13385704; doi:10.3389/fphar.2026.1819017)
Supplement: Supplementary file 1 [file Supplementaryfile1.docx]

Supplementary Material


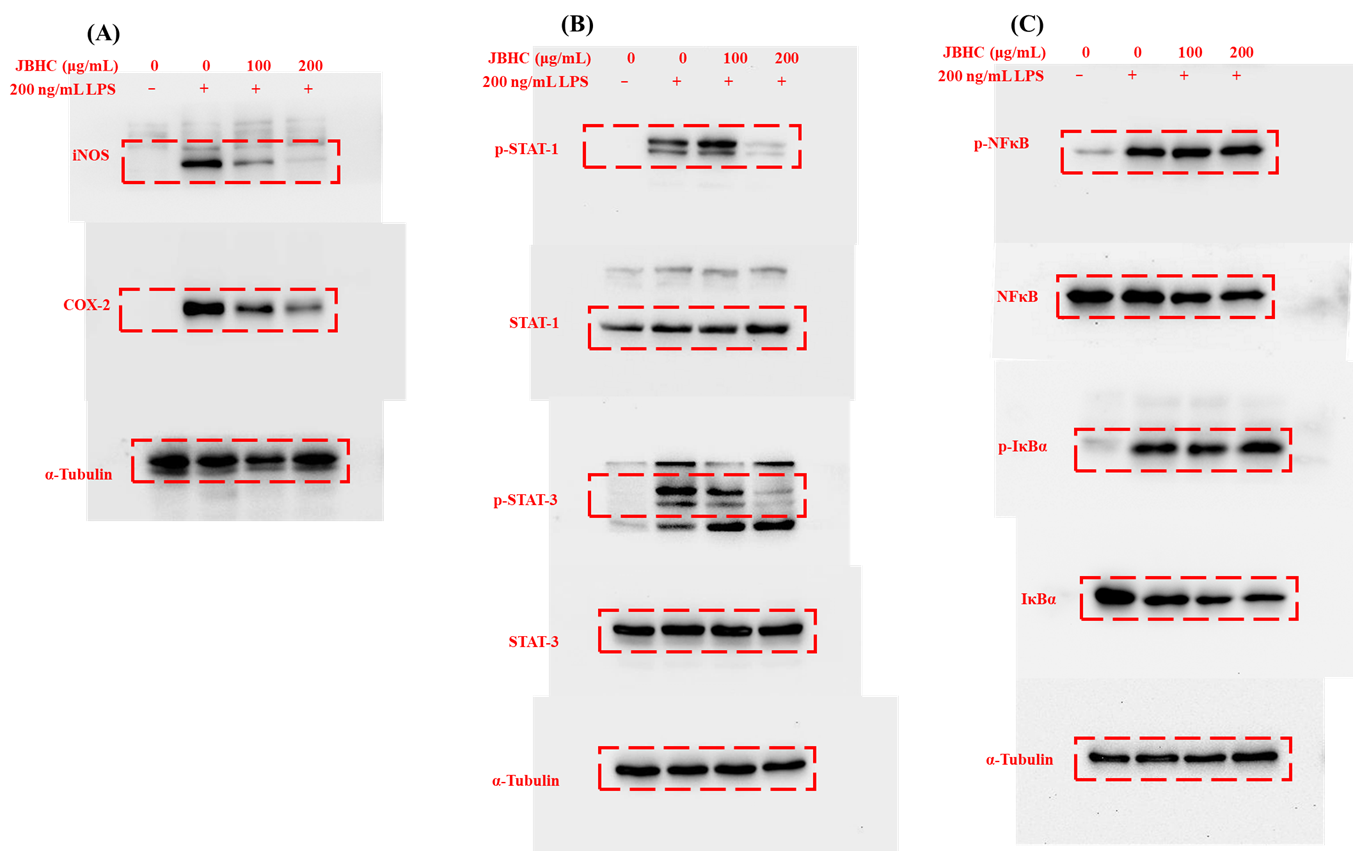


**Supplementary Figure 1.** **Western blot whole band in Figure 3**

**(A)** Figure 3J. **(B)** Figure 3M. **(C)** Figure 3N.


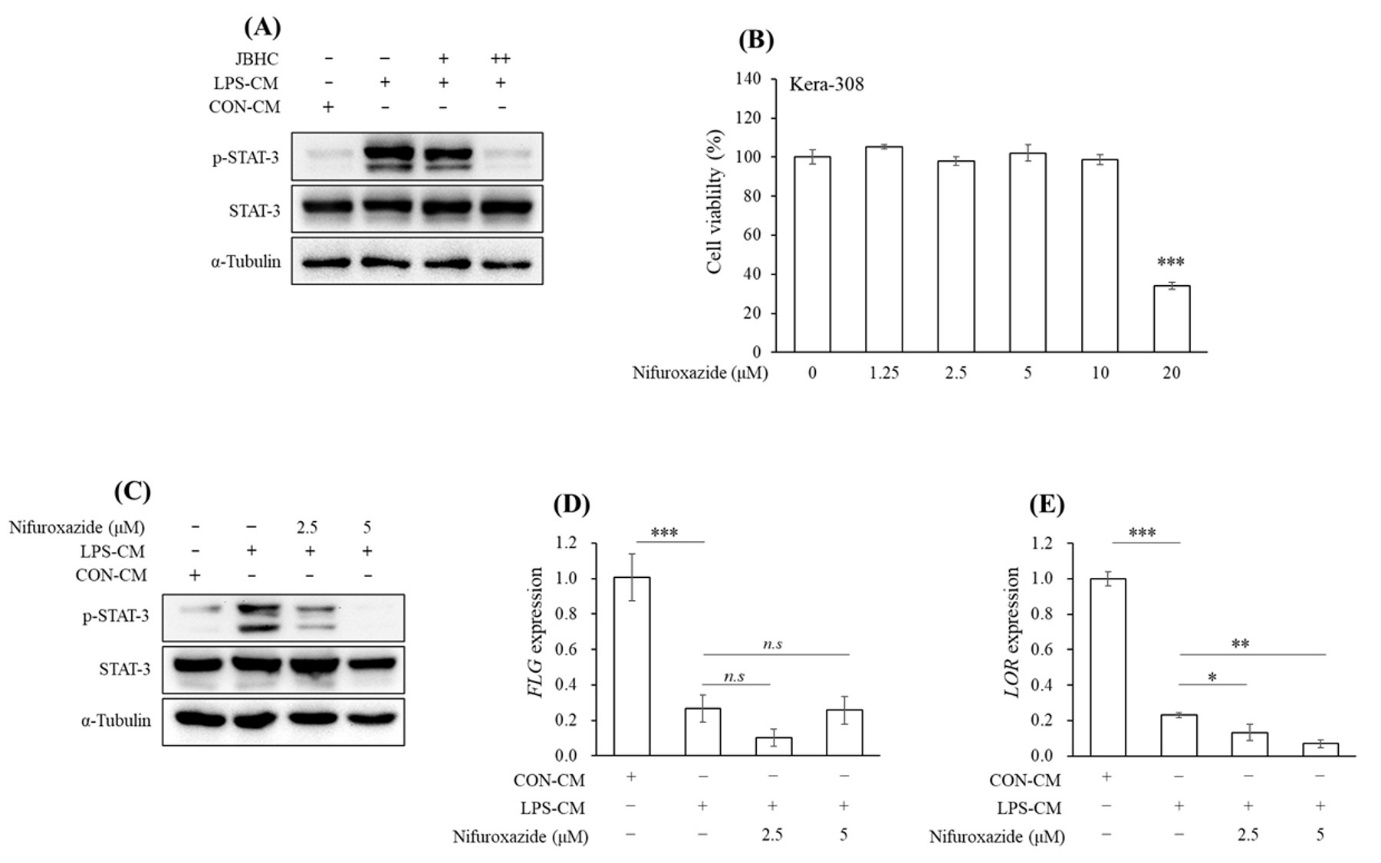


**Supplementary Figure 2. STAT3 inhibition does not restore keratinocyte barrier gene expression under psoriatic inflammatory stress**

**(A)** Kera308 cells were treated with each CM for 24 h. Protein expression of STAT3 was determined by western blot. **(B)** Cytotoxicity of Nifuroxazide in Kera308 cells after 24 h of treatment, as evaluated by MTT. **(C)** Kera308 cells were treated with each CM for 24 h. Protein expression of STAT3 was determined by western blot. **(D, E)** Kera308 cells were treated with each CM for 24 h. mRNA expression of FLG **(D)** and LOR **(E)** was analyzed by qPCR. Data are presented as the mean ± SD from at least three independent experiments. Statistical significance was determined at *p* < 0.05.

**Supplementary Table 1. The list of primary antibodies**

| **Antibody** | **Catalog No** | **Company** |
| --- | --- | --- |
| p-NFκB | #3033 | Cell Signaling Technology |
| p-IκBα | #2859 |  |
| IκBα | #4814 |  |
| p-STAT-1 | #9167 |  |
| STAT-1 | #9172 |  |
| p-STAT-3 | #9145 |  |
| STAT-3 | #30835 |  |
| COX-2 | #12282 |  |
| iNOS | PA1-036 | Invitrogen |
| NFκB | sc-8008 | Santa Cruz Biotechnology |

**Supplementary Table2. Detailed HPLC condition**

| **Column** | Agilent Eclipse XDB-C18(4.6 x 250mm, 5μm) | | |
| --- | --- | --- | --- |
| **Column temp.** | 30℃ | | |
| **Detector** | UV-Vis | | |
| **Wavelength** | 380nm | | |
| **Flowrate** | 1.0 mL/min | | |
| **Injection vol.**  **and dose** | 20 μL of 100 mg/ml samples dissolved in MeOH | | |
| **Solvent** | A: DIW  B: Methanol | | |
| **Gradient**  **condition** | Time (min) | A % | B % |
|  | 0 | 55 | 45 |
|  | 23 | 30 | 70 |
|  | 23.1 | 0 | 100 |
|  | 30 | 0 | 100 |
|  | 30.1 | 55 | 45 |
|  | 40 | 55 | 45 |

**Supplementary Table 3. The sequence of qPCR primers**

| **Gene name** |  | **Sequence ( 5' → 3' )** |
| --- | --- | --- |
| ***NOS2*** | Forward | CAGCACAGGAAATGTTTCAGC |
|  | Reverse | TAGCCAGCGTACCGGATGA |
| ***PTGS2*** | Forward | TTTGGTCTGGTGCCTGGTC |
|  | Reverse | CTGCTGGTTTGGAATAGTTGCTC |
| ***IL1A*** | Forward | ACGGCTGAGTTTCAGTGAGACC |
|  | Reverse | CACTCTGGTAGGTGTAAGGTGC |
| ***IL1B*** | Forward | TTGACGGACCCCAAAAGATG |
|  | Reverse | AGAAGGTGCTCATGTCCTCA |
| ***IL6*** | Forward | GGTGACAACCACGGCCTTCCC |
|  | Reverse | AAGCCTCCGACTTGTGAAGTGGT |
| ***TNFA*** | Forward | TATGGCTCAGGGTCCAACTC |
|  | Reverse | CTCCCTTTGCAGAACTCAGG |
| ***CCL2*** | Forward | CAAGATGATCCCAATGAGTAG |
|  | Reverse | TTGGTGACAAAAACTACAGC |
| ***CXCL2*** | Forward | TCCAGGTCAGTTAGCCTTGC |
|  | Reverse | CGGTCAAAAAGTTTGCCTTG |
| ***CXCL10*** | Forward | CAGTGAGAATGAGGGCCATAGG |
|  | Reverse | CGGATTCAGACATCTCTGCTCAT |
| ***CXCL11*** | Forward | CCGAGTAACGGCTGCGACAAAG |
|  | Reverse | CCTGCATTATGAGGCGAGCTTG |
| ***CSF3*** | Forward | TTGGCAACATCCAGCTGAAG |
|  | Reverse | GCAGGCTCTATCGGGTATTTCC |
| ***ICAM1*** | Forward | CAATTTCTCATGCCGCACAG |
|  | Reverse | AGCTGGAAGATCGAAAGTCCG |
| ***FLG*** | Forward | GTTTCCAAACACATGGATCAAAT |
|  | Reverse | TTTGAATCTTGTTGGTGTCTGTG |
| ***LOR*** | Forward | CCTGTGGGTTGTGGAAAGACC |
|  | Reverse | AGAGCCTCCTCCAGATGAGC |
| ***ACTB*** | Forward | TGTCCACCTTCCAGCAGATGT |
|  | Reverse | AGCTCAGTAACAGTCCGCCTAGA |

**Supplementary Table 4. HPLC peak number of LE70E**

| **Peak No.** | **Ret time (min)** | **Area** | **Heigh** |
| --- | --- | --- | --- |
| 1 | 2.315 | 938.49597 | 83.6351 |
| 2 | 2.798 | 62.93536 | 4.4252 |
| 3 | 3.694 | 459.57025 | 10.6758 |
| 4 | 4.147 | 113.19573 | 8.5587 |
| 5 | 4.436 | 21.98349 | 2.1735 |
| 6 | 4.741 | 2.13648 | 0.2730 |
| 7 | 5.429 | 38.05778 | 3.8349 |
| 8 | 5.673 | 201.70367 | 17.5709 |
| 9 | 6.19 | 9.39353 | 0.3659 |
| 10 | 7.57 | 32.16696 | 0.7115 |
| 11 | 8.631 | 4.25652 | 0.2112 |
| 12 | 9.372 | 2.91749 | 0.1497 |
| 13 | 10.03 | 5.01063 | 0.2213 |
| 14 | 12.105 | 14.57727 | 0.7571 |
| 15 | 21.094 | 2.74748 | 0.1075 |
| 16 | 26.643 | 203.87796 | 6.4646 |
| 17 | 26.803 | 21.01035 | 3.2029 |
| 18 | 26.979 | 9.03476 | 1.6558 |
| 19 | 27.243 | 11.86393 | 1.8935 |
| 20 | 27.391 | 32.23645 | 3.9263 |
| 21 | 27.913 | 89.23033 | 8.1990 |
| 22 | 28.089 | 111.04966 | 9.7130 |
| 23 | 28.258 | 88.94688 | 9.9243 |
| 24 | 28.446 | 10.84947 | 1.2793 |
| 25 | 28.722 | 119.06729 | 14.1140 |
| 26 | 28.961 | 23.8067 | 3.3786 |
| 27 | 29.258 | 100.15261 | 9.3884 |
| 28 | 29.686 | 57.19409 | 3.5908 |
| 29 | 30.107 | 73.47599 | 2.6541 |
| 30 | 30.951 | 50.69378 | 2.1801 |
| 31 | 31.293 | 44.68729 | 2.3527 |
| 32 | 31.586 | 61.30951 | 2.6411 |
| 33 | 32.031 | 92.38952 | 3.4272 |
| 34 | 32.457 | 86.56548 | 3.9694 |
| 35 | 33.043 | 172.26518 | 5.2849 |
| 36 | 33.434 | 72.2796 | 8.4853 |

**Supplementary Table 5. HPLC peak number of AG70E**

| **Peak No.** | **Ret time (min)** | **Area** | **Heigh** |
| --- | --- | --- | --- |
| 1 | 2.066 | 21.24553 | 2.94710 |
| 2 | 2.342 | 1244.10645 | 98.55968 |
| 3 | 2.886 | 408.76318 | 16.74245 |
| 4 | 3.841 | 7.81797 | 0.62515 |
| 5 | 4.249 | 1.77296 | 0.17380 |
| 6 | 4.714 | 2.45175 | 0.29634 |
| 7 | 5.014 | 27.03711 | 2.08884 |
| 8 | 5.382 | 34.45528 | 1.53111 |
| 9 | 5.707 | 18.41452 | 1.57669 |
| 10 | 6.172 | 161.50012 | 10.60448 |
| 11 | 6.52 | 6.07454 | 0.19976 |
| 12 | 7.792 | 36.44853 | 1.10920 |
| 13 | 8.363 | 25.85859 | 0.69728 |
| 14 | 9.437 | 13.35838 | 0.52072 |
| 15 | 10.136 | 38.75915 | 2.43955 |
| 16 | 10.964 | 33.71109 | 1.50741 |
| 17 | 11.492 | 7.28077 | 0.36965 |
| 18 | 12.845 | 2.43158 | 0.06792 |
| 19 | 13.72 | 19.31535 | 0.98135 |
| 20 | 14.492 | 19.91180 | 1.11818 |
| 21 | 15.512 | 5.39529 | 0.14779 |
| 22 | 16.892 | 2.85280 | 0.13947 |
| 23 | 17.749 | 2.30411 | 0.09162 |
| 24 | 18.224 | 8.57559 | 0.20686 |
| 25 | 19.293 | 6.06413 | 0.27400 |
| 26 | 19.83 | 15.49405 | 0.87853 |
| 27 | 20.321 | 6.54035 | 0.37980 |
| 28 | 20.749 | 97.13603 | 5.21022 |
| 29 | 22.04 | 13.54129 | 0.64456 |
| 30 | 22.723 | 2.65348 | 0.13692 |
| 31 | 23.522 | 2.64171 | 0.15031 |
| 32 | 24.035 | 5.71149 | 0.26105 |
| 33 | 24.92 | 61.55207 | 4.19651 |
| 34 | 26.765 | 2157.65723 | 37.12329 |
| 35 | 28.007 | 178.35158 | 9.31522 |
| 36 | 28.348 | 92.47778 | 6.75123 |
| 37 | 28.639 | 165.70146 | 9.22113 |
| 38 | 29.378 | 63.64280 | 3.83144 |
| 39 | 30.426 | 84.19093 | 2.24609 |
| 40 | 30.696 | 36.24885 | 2.42549 |
| 41 | 31.231 | 81.10843 | 2.82587 |
| 42 | 32.526 | 287.65833 | 3.70609 |
| 43 | 32.951 | 93.63361 | 3.76829 |
| 44 | 33.445 | 64.86275 | 6.97783 |

**Supplementary Table 6. HPLC peak number of AM70E**

| **Peak No.** | **Ret time (min)** | **Area** | **Heigh** |
| --- | --- | --- | --- |
| 1 | 2.28100 | 374.00165 | 30.60694 |
| 2 | 2.80600 | 109.74400 | 5.16617 |
| 3 | 3.40000 | 64.31205 | 2.97963 |
| 4 | 3.90600 | 10.15345 | 0.69692 |
| 5 | 4.41700 | 5.91082 | 0.46261 |
| 6 | 5.12100 | 1.94075 | 0.09887 |
| 7 | 5.48900 | 2.76607 | 0.20580 |
| 8 | 5.98500 | 19.19824 | 0.49053 |
| 9 | 7.61700 | 9.74258 | 0.24579 |
| 10 | 8.59200 | 1.60679 | 0.09974 |
| 11 | 9.38700 | 11.35984 | 0.43270 |
| 12 | 10.38300 | 4.83062 | 0.24554 |
| 13 | 11.66000 | 11.14073 | 0.33356 |
| 14 | 13.32800 | 6.00195 | 0.24827 |
| 15 | 13.76200 | 9.54365 | 0.45648 |
| 16 | 16.63100 | 135.69217 | 5.35677 |
| 17 | 18.25100 | 2.42622 | 0.14710 |
| 18 | 18.98000 | 7.42330 | 0.22172 |
| 19 | 20.70600 | 3.58768 | 0.19230 |
| 20 | 22.26600 | 17.14138 | 0.83867 |
| 21 | 26.63700 | 814.24670 | 16.90206 |
| 22 | 28.64500 | 32.83172 | 1.16048 |
| 23 | 29.31800 | 13.91441 | 0.46014 |
| 24 | 30.17500 | 1.53424 | 0.12350 |
| 25 | 30.59100 | 7.09600 | 0.54642 |
| 26 | 33.45200 | 35.06378 | 4.60841 |
